# Growth ability, carbon source utilization and biochemical features of the new specie *Zalaria obscura*

**DOI:** 10.1007/s11274-022-03417-y

**Published:** 2022-09-23

**Authors:** Raffaella Campana, Francesco Palma, Maurizio Sisti

**Affiliations:** 1 Department of Biomolecular Sciences, University of Urbino Carlo Bo, Urbino, Italy; 2 Division of Pharmacology and Hygiene, Department of Biomolecular Sciences, University of Urbino Carlo Bo, Via S. Chiara 27, 61029 Urbino, Italy

**Keywords:** Biochemical profile, Growth characteristics, Nutritional requirements, Pigments, *Zalaria obscura*

## Abstract

This research investigated the characteristics of *Zalaria obscura* LS31012019 in terms of growth ability in different media (SDB, YPD and TSB) and temperatures (22, 25 and 37 °C), utilization of several carbon sources (Glucose, Fructose, Lactose, Sucrose, Xylose, Glycerol and Mannitol at 5, 2 and 1%) and several biochemical features (total protein content, Glutathione, pigments), in comparison with those of the phylogenetically related *Aureobasidium pullulans* ATCC 15233. The best growth of *Z. obscura* LS31012019 was obtained in YPD at 25 °C with the highest OD value (0.45) after 144 h of incubation, similar to that of *A. pullulans* ATCC 15233 (0.48). Glucose resulted the preferred carbon source for both the considered yeasts but also sucrose resulted in efficacy supporting the growth of *Z. obscura* LS31012019 and *A. pullulans* ATCC 15233, for their ability in converting sucrose to glucose and fructose and the latter into glucose. Interestingly, *Z. obscura* LS31012019 utilized also glycerol and mannitol. The biochemical analysis showed the similarity of protein profile in *Z. obscura* LS31012019 and *A. pullulans* ATCC 15233 (from 90 to 20 kDa) and a reduced GSH content (0.321 and 0.233 µmol/mg). The pigments extraction with hexane generated a yellow oleaginous pellet in both the strains, while a yellow solid matrix more intensely coloured in *A. pullulans* ATTC 15233 was visible with the following solvent extractions. Overall, our data showed that *Z. obscura* LS31012019 can grow in different media and temperatures and utilize carbon sources apart from glucose and sucrose, shifting to a non-fermentative metabolism. These results improve the information regarding the characteristics of *Z. obscura*, opening a new field of investigation for the possible application of new species of black yeasts in human application.

## Introduction

*Zalaria obscura* belongs to the new genus *Zalaria*, family *Zalariaceae* as introduced by Humpries and collaborators in 2017. Few investigations are still present in the literature and poor information is available on the specific characteristics of this yeast. As regards the growth on solid media, the colonies appear cream-coloured, red-brown, olive-brown, dark brown, or black, often covered in slimy masses of conidia or yeast-like cells. From a microscopic point of view, hyphae are transversely and longitudinally septate, hyaline and thin-walled when young, becoming melanized and thick-walled with ageing. Chlamydospores are dark brown, smooth to lightly rough-walled, globose to ellipsoidal and septate, while conidia are often yeast-like and appear hyaline, aseptate, smooth-walled, ellipsoidal to lemon-shaped and rather variable in size (Humpries et al. 2017). In 2020, our research group have isolated a particular fungal strain from a damaged wooden sculpture, successively identified by the molecular method as *Zalaria obscura* (named *Z. obscura* LS31012019) (Sabatini et al. 2020). This microorganism has several common traits with *Aureobasidium* spp. in terms of colony morphology, growth conditions and presence of melanized cells. Indeed, both were classified as black yeasts, a *terminus technicus* indicating a group of fungi that, taxonomically and phylogenetically is quite heterogeneous, but having in common the melanized cell walls and the formation of daughter cells by yeast-like multilateral or polar budding (Sterflinger 2006). In general, black yeasts are polyextremotolerant, able of colonizing a wide range of extreme and hostile environments for their intrinsic ability to survive under acidic, alkaline, and toxic conditions, and to tolerate high temperatures, low nutrient availability, osmotic and mechanical stresses. Occasionally, some species can cause human infections (Moreno et al. 2018).

A particularly interesting aspect in the survival of black yeasts in harsh conditions is related to the presence of pigments, such as melanins and carotenoids. These pigments, deposited in the cell walls of melanized fungi, are not essential for growth and development but play an important role in survival ability and virulence (Seyedmousavi et al. 2014), acting as a protection against solar irradiation, temperature variation, and many environmental stresses. Melanins, negatively charged hydrophobic pigments of high molecular weight, are composed by polymerized phenolic or/and indolic compounds. In fungal cells are found two main types of melanin, the dihydroxynaphthalene (DHN)-melanin of *Aspergillus* species and black fungal pathogens and the 3,4-dihydroxyphenylalamine (DOPA)-melanin found in *Cryptococcus neoformans* (Gow et al. 2017; Nosanchuk et al. 2015). Carotenoids, terpenoid pigments of yellow, orange, and red colour synthesized in species from all taxa exception of animals (Goldwin 1980), are not essential for fungi and are accumulated in much lower concentrations compared to plants and algae. Carotenoids are found in all fungal groups along with non-carotenogenic species (Sandman 2022; Valadon 1976), offering a structural diversity compared to those found elsewhere. Moreover, black yeasts can produce different kinds of extracellular enzymes for utilizing a wide range of substrates as carbon and nitrogen sources for their cell growth and metabolisms (Chi et al. 2022). Also in this case, all the above mentioned information can be referred to *Aureobasidium* spp., but no data are presented for *Zalaria* species. The lack of knowledge of metabolic, nutritional requirements or any type of biochemical profiles, let us to better characterize the growth ability, as well as define several biochemical aspects of our *Z. obscura* LS31012019 isolate, in comparison to the phylogenetically related *Aureobasidium pullulans*. For this, the protein profile, the ability to grow in different culture media and temperatures, the utilization of different carbon sources as well as the quantification of glutathione (GSH) and the extraction of pigments were carried out.

## Material and methods

### Fungal strains and culture conditions

*Zalaria obscura* LS31012019 and *Aureobasidium pullulans* ATCC 15233 were used in this study. The strains were grown on Potato Dextrose Agar (PDA, Liofilchem, Italy) at 25 °C for 5–7 days. The fungal suspensions were prepared following the indication of the National Committee for Clinical Laboratory Standards (NCCLS 2008). Briefly, for each strain, one or two colonies were harvested from PDA plate and diluted in sterile 0.85% saline solution with 0.05% Tween 80. The suspension was mixed for 15 s and adjusted to an optical density at 530 nm corresponding to about 10^6^ cells per mL. Moreover, each inoculum was quantified with the agar plate count method on PDA.

### Polyacrylamide gel electrophoresis (SDS-PAGE)

In this case, the strains were grown in liquid Yeast Nitrogen Base (YNB, Sigma, Milan, Italy) containing 50 g/L of sucrose and chloramphenicol at 25 °C per 5 days. A pre-inoculum preceded this step from a single colony in the same medium. The biomass was harvested by centrifugation at 15,000×*g* for 20 min at 4 °C, and the pellet was washed with water twice by re-suspension/centrifugation cycle. Washed cells were lyophilized, and the dried-material weight and suspended in NaHCO_3_ 0.2 M pH 9.0 in a ratio of 0.1 g/mL. Cell-lysis was achieved by sonication with a sonicator Labsonic (Braun) set to a power of 200 W, a duty cycle of 0.5 and a probe of 30 cm. We proceeded with two pulses of 90 s interspersed with a pause, placing the sample in an ice bath. The suspension was transferred in a 50 mL tube and centrifuged at 5000 rpm for 30 min in a refrigerated centrifuge. Clear supernatant was transferred into a fresh tube, used as fungal cell lysate (FCL), and stored at − 20 °C. Total protein concentration was assayed by Bio-Rad protein assay kit, based on the Bradford dye-binding method (Bradford 1976), and BSA for the calibration curve. Briefly, the supernatant was diluted 1:10, with water, and then 10, 25, and 50 µL were processed as described in the instruction manual. A volume of lysate corresponding to 30 µg of protein was lyophilized; then, the pellet was resuspended in 30 µL of Sample buffer (2.0%, w/v) SDS, 10% (v/v) glycerol, 0.01% (w/w) bromophenol blue, 50 mM Tris–HCl pH 6.8 and 3.0% (v/v) β-mercaptoethanol), denatured by boiling for 5 min at 95 °C, and loaded onto an SDS-PAGE gel (5.0% stacking, 12% resolving). After electrophoresis at 100 V for approximately 1 h, the gel was stained with Coomassie Brilliant Blue R-250 (Sigma-Aldrich, B8647). Molecular masses of bands were estimated using the ECL Rainbow Marker–High Range (Amersham, RPN756E).

### Growth ability in different liquid media and temperatures

The ability *Z. obscura* LS31012019, in comparison to *A. pullulans* ATCC 15233, to grow in different liquid media at different temperatures was determined. For this, the right amount of each yeast suspension, obtained as described above, was transferred in sterile tubes containing 5 mL of Sabouraud Dextrose Broth (SDB; Sigma, Milan, Italy), YPD (yeast extract 10.0 g/L, polypeptone 20.0 g/L, dextrose 20.0 g/L) or Tryptone Soy Broth (TSB; VWR, Milan, Italy) to obtain a final concentration about 10^4^ cell/mL. The composition of each broth was presented in Table 1.

**Table 1:** Chemical composition of the liquid media used in this study for growth ability assay

Liquid media	Manufacturer	Chemical composition
Yeast extract peptone dextrose broth (YPD)	Prepared in our laboratory	Yeast extract 10 g/L
		Polypeptone 20 g/L
		Dextrose 20 g/L
		pH 5.5 ± 0.2 at 25 °C
Sabouraud dextrose broth (SDB)	Sigma, Milan, Italy	Mycological peptone 10 g/L
		Dextrose 20 g/L
		pH 5.6 ± 0.2 at 25 °C
Tryptone soya broth (TSB)	Oxoid, Milan, Italy	Pancreatic digest of casein 17 g/L
		Enzymatic digest of soya bean 3 g/L
		Di-potassium hydrogen phosphate 2.5 g/L
		Glucose 2.5 g/L
		NaCl 5 g/L
		pH 7.3 ± 0.2 at 25 °C

The inoculated tubes were divided as in the following scheme:

- 6 tubes (2 with SDB, 2 with YPD, 2 with TSB) were incubated at 37 °C;
- 6 tubes (2 with SDB, 2 with YPD, 2 with TSB,) were incubated at 25 °C;
- 6 tubes (2 with SDB, 2 with YPD, 2 with TSB) were incubated at 22 °C (considered as room temperature, RT).

For each condition, three tubes of the different not inoculated broths as well as two tubes of inoculated YP (yeast extract 10.0 g/L, polypeptone 20.0 g/L), were left as controls. The tubes were checked every day up to 7–10 days and the ability to grow was daily assessed by optical density at 530 nm. All treatments were performed in triplicate and all the data were the average of independent cultures.

### Growth ability in presence of different carbon sources

The ability *Z. obscura* LS31012019, in comparison to *A. pullulans* ATCC 15233, to grow in a liquid medium with different sources of carbon (Glucose, Fructose, Lactose, Sucrose, Xylose, Glycerol and Mannitol) (Sigma) was determined, as decribed by Santos et al. (2021) with several modifications. Each yeast suspension, obtained as described above, was resuspended in YP to about 10^4^ cells/mL; then, 100 µL of each suspension was transferred in a 96-well plate containing 100 µL of YP with each carbon source (named YPG, YPF, YPL, YPS, YPX, YPGL, YPM) at 5%, 2% and 1%. Wells containing only YP (not inoculated) were used as negative controls. Plates were incubated at 25 °C up to 12 days and exanimated every 48 h by spectrophotometer multi-reader Spark (TECAN, Switzerland) equipped with Magellan software for data analysis. Each experiment was performed in triplicate using independent cultures.

### GSH assay

GSH levels were measured using Ellman’s method (Ellman 1959). Within 2 h after preparation, the FCL was subjected to the GSH assay. Twenty microliters of suitably-diluted sample, 780 µL of 0.2 M sodium bicarbonate at pH 9.0 and 80 µL of 0.2 g/L of DTNB (5,5′-dithiobis-[2-nitrobenzoic acid]) in 1% (w/v) sodium citrate were placed in a one-milliliter plastic cuvette. After a 15 min incubation in the dark, the absorption was read at 412 nm in a Beckman DU 7500 spectrophotometer at room temperature. Quantitative determination was performed by converting the absorbance value through a fresh calibration curve generated using GSH standards ranging from 0 to 1 mM, and the final value was expressed as µmol of GSH/mg of total protein.

### Extraction of fungal pigments

A colony of either *Z. obscura* LS31012019 or *A. pullulans* ATCC 15233 was transferred from PDA plates to 250 mL Erlenmeyer flasks containing 50 mL of semi-synthetic medium (50 g/L sucrose, 2.0 g/L yeast extract, 5.0 g/L KH_2_PO_4_, 0.2 g/L MgSO_4_ × 7 H_2_O, 1.0 g/L NaCl, and 0.01 g/L FeSO_4_ × 7 H_2_O) and chloramphenicol (50 µL of a 30 mg/mL of ethanol solution). Then, each culture was incubated for 5 days at 25 °C. The yeast cells were harvested by centrifugation at 15,000×*g* for 20 min at 4 °C. The lyophilized washed cells (obtained as described above in paragraph 2.2) were ground to a fine powder in a mortar and pestle; then, 5 mL of hexane were added, and the suspension was transferred in a fresh Falcon tube and incubated at room temperature for 30 min. The supernatant was moved into a new 50 mL test tube after centrifugation at 4800 rpm for 10 min in a Beckman centrifuge GS-15R. Five millilitres of absolute ethanol was added to the pellet, dried under a hood, and centrifuged again after 30 min of incubation. The extraction with ethanol/1% acetic acid (v/v) 80:20 was performed as a last extraction following the upper procedure. The supernatants collected from hexane, absolute ethanol, and acid ethanol extraction were dried under a vacuum and were subjected to image analysis.

## Results

### Electrophoretic profile of *Z. obscura* LS31012019

**Fig. 1:**
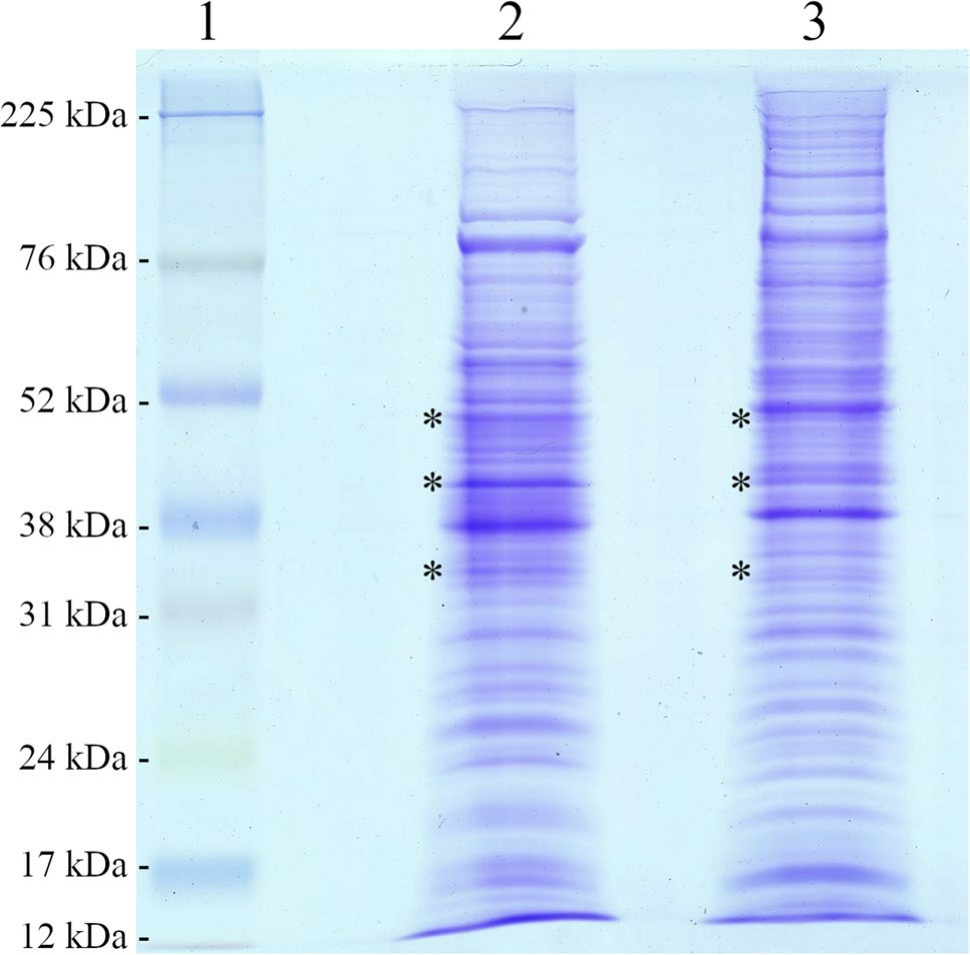
SDS-PAGE of fungal cell lysate proteins produced by *A. pullulans* ATCC 15233 and *Z. obscura* LS31012019. Lane 1: protein marker; lane 2: *A. pullulans* ATCC 15233; lane 3: *Z. obscura* LS31012019. The asterisks indicate bands with an equal molecular weight (Mw), which could differ in intensity

The value of the total content of protein lysate of *Z. obscura* LS31012019 (2.96 ± 0.27 mg/mL) as well as the one of *A. pullulans* ATCC 15233 (6.58 ± 0.41 mg/mL) (Table 2) was used to determine the sample volume to be loaded onto the electrophoresis. Figure 1 reports the SDS-PAGE of the total protein of *Z. obscura* LS31012019 compared to that of *A. pullulans* ATCC 15233. As shown, the two electrophoretic patterns show the absence of identical bands but a similar profile ranging from 90 to 20 kDa. By comparing the two electrophoretic profiles, we could identify a few protein bands with an equal molecular weight that differ in intensity.

**Table 2:** Total protein content in *Z. obscura* LS31012019 and *A. pullulans* ATCC 15233 cell lysates

Yeast strains	Protein content in cell lysate (mg/mL)
*Z. obscura* LS31012019	2.96 ± 0.27
*A. pullulans* ATCC 15233	6.58 ± 0.41

### Growth abilities in liquid media at different temperatures

**Fig. 2:**
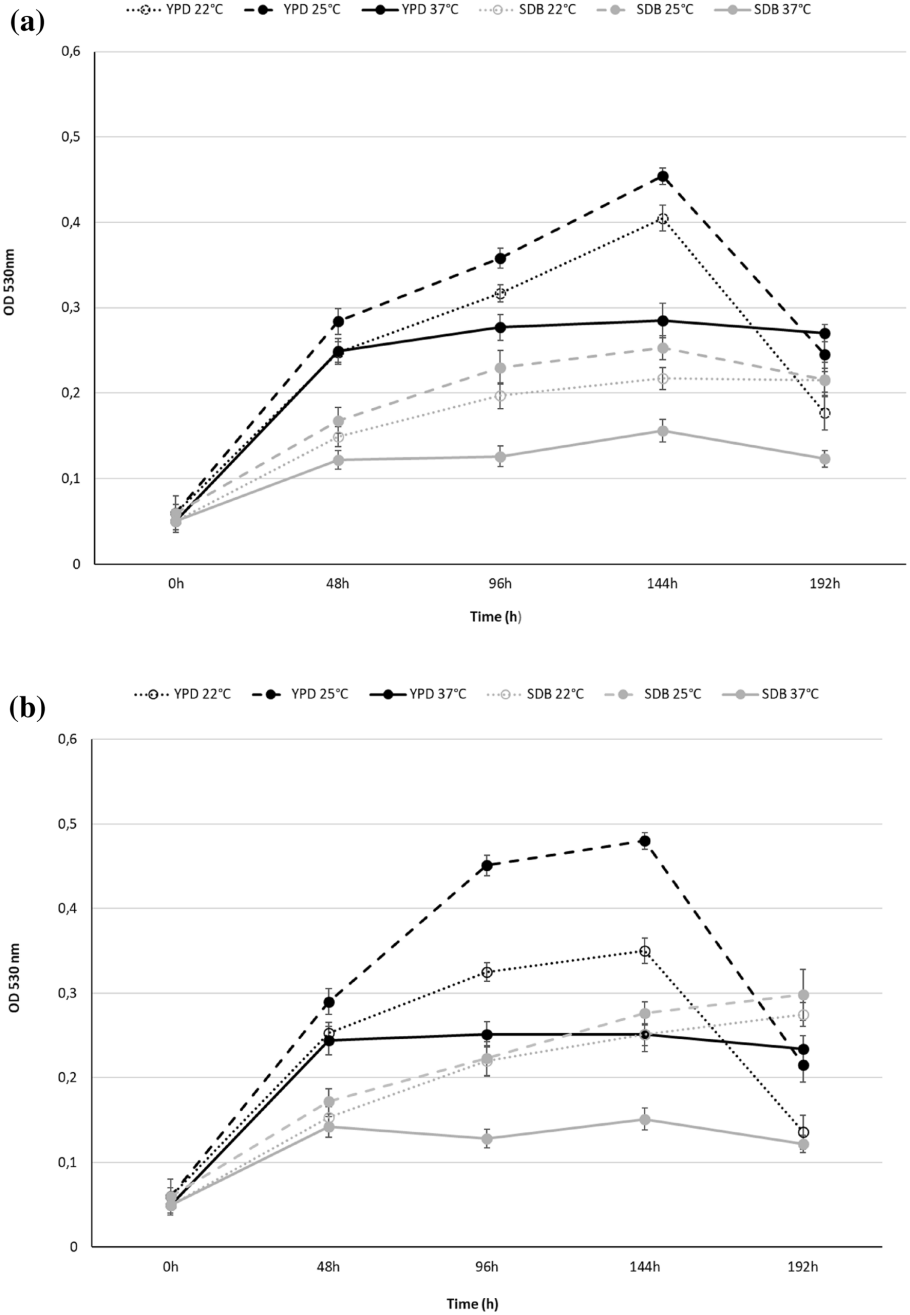
Growth kinetics *Z. obscura* LS31012019 **a** in comparison to *A. pullulans* ATCC 15233 **b** in different media and temperatures. Values are expressed as the means ± standard deviations

**Fig. 3:**
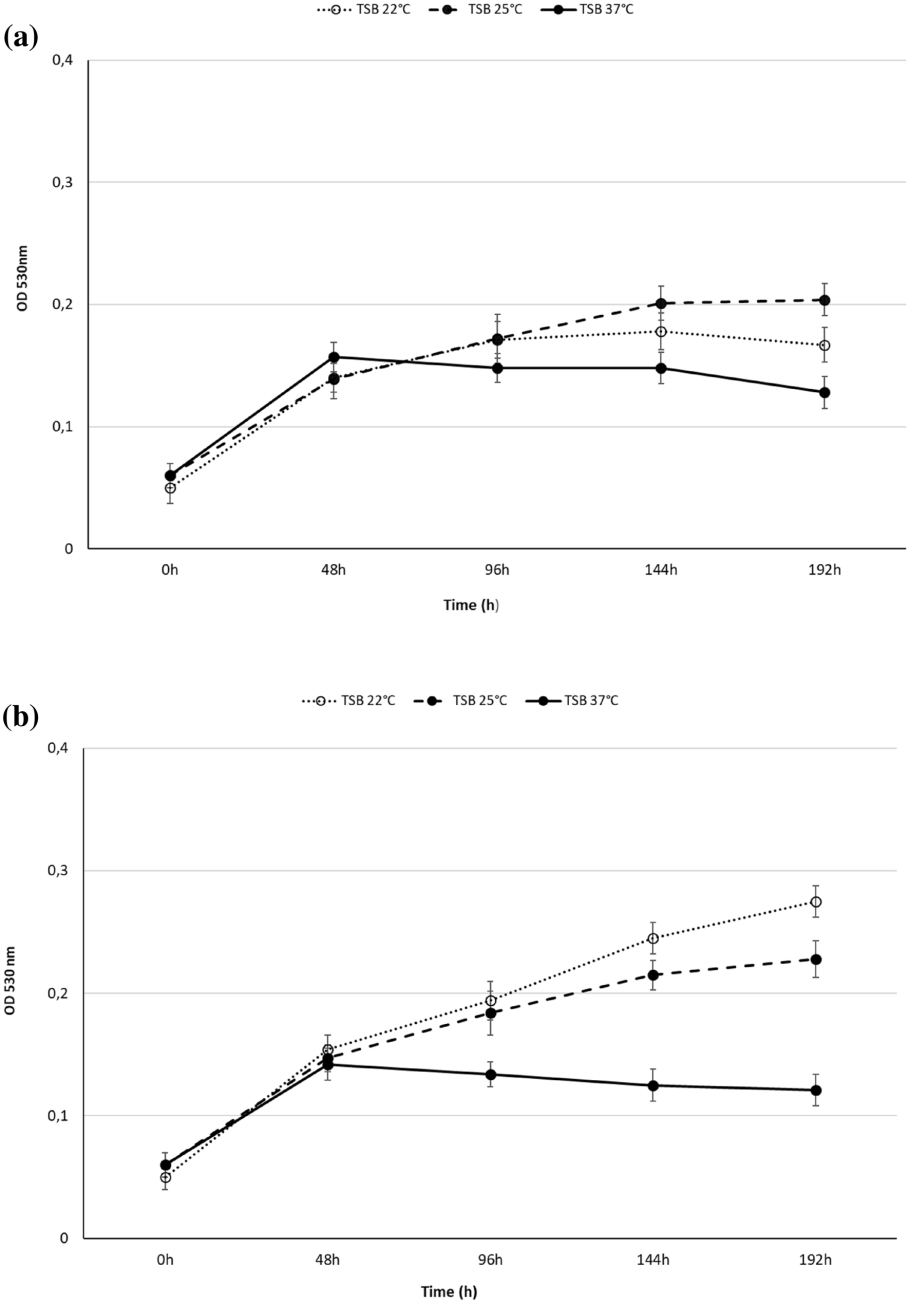
Growth kinetics of *Z. obscura* LS31012019 **a** in comparison to *A. pullulans* ATCC 15233 **b** in TSB at different temperatures. Values are expressed as the means ± standard deviations

The growth ability of *Z. obscura* LS31012019, in comparison to *A. pullulans* ATCC 15233, in different liquid media was examined. Data revealed that YPD was the most suitable medium for *Z. obscura* LS31012019 and *A. pullulans* ATCC 15233 growth at each selected temperature with the best performance at 25 °C (Fig. 2), while more limited growth rates were evidenced in TSB (Fig. 3), medium with a pH 7.2 and lower glucose concentration (Table 1).

In detail, after 144 h of incubation in YPD at 25 °C, the highest OD value of 0.45 ± 0.01 was reached for *Z. obscura* LS31012019 compared to 0.25 ± 0.013 obtained in SDB under the same conditions (Fig. 2a); similarly, the highest growth rate of 0.48 ± 0.01 was observed for *A. pullulans* ATCC 15233 in YPD at 25 °C for 144 h in comparison to 0.276 ± 0.014 in SBD (Fig. 2b). When the incubation was performed in YPD at 22 °C, low OD values were observed for *Z. obscura* LS31012019 (maximum OD 0.405 ± 0.01 after 144 h), as well as for *A. pullulans* ATCC 15233 (maximum OD 0.35 ± 0.015 after 144 h), during all the experiment (Fig. 2a, b). For both the strains, a drastically growth decrease was noted after 192 h of incubation, showing ODs of 0.245 ± 0.02 and 0.177 ± 0.02 in YPD at 25 °C and 22 °C respectively in the case of *Z. obscura* LS31012019; similarly, ODs of 0.215 ± 0.02 and 0.137 ± 0.2 were registered for *A. pullulans* ATCC 15233 in YPD at 25 °C and 22 °C. On the contrary, the incubation in YPD at 37 °C reached, for both the strains, lower growth rates (below OD 0.3), without evidencing the rapid decrease of OD values observed in the other two culture conditions. As expected, both the strains were able to grow in YP without any additional sugar, but slighter compared to YPD (data not shown).

### Carbon source utilization

**Fig. 4:**
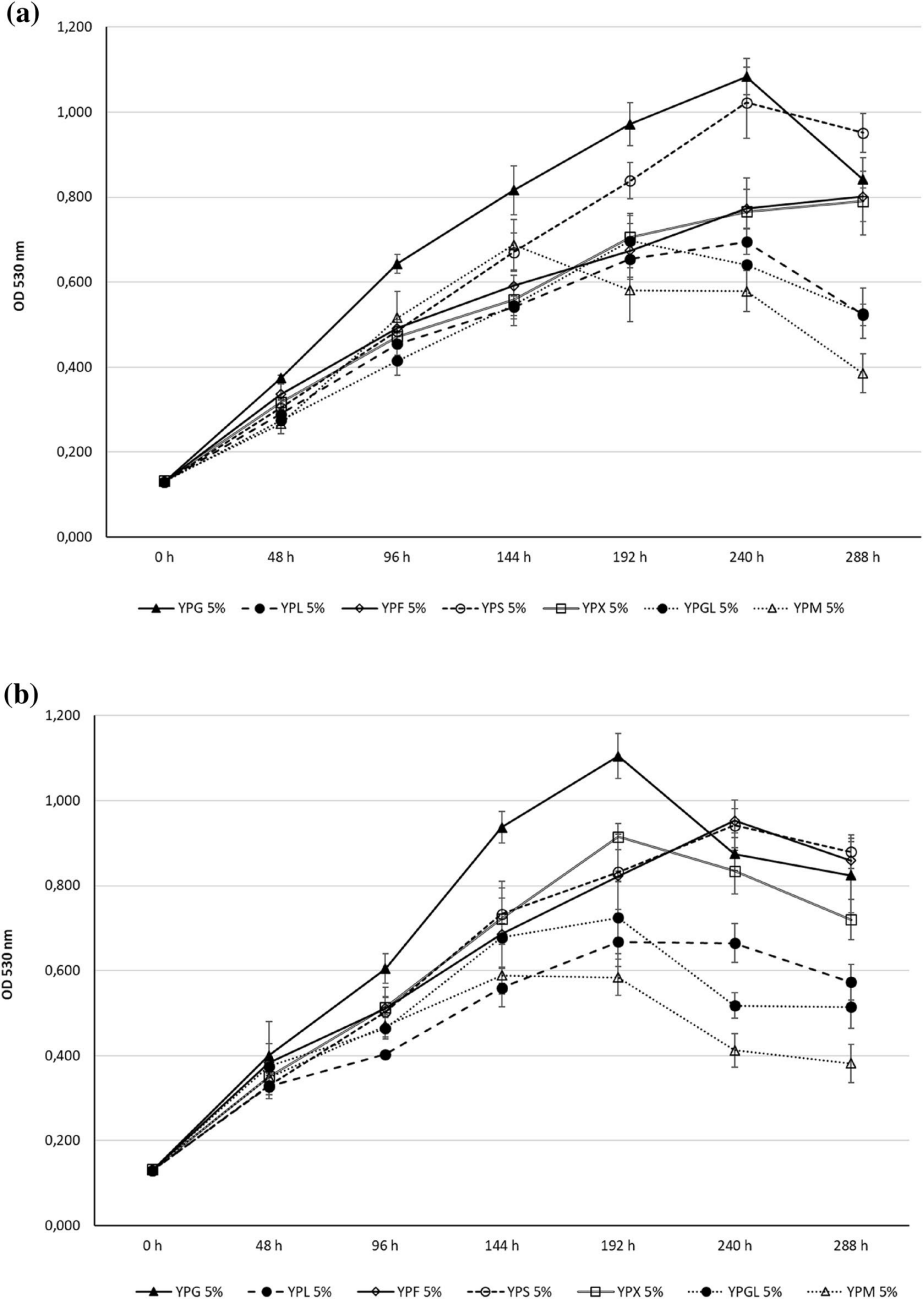

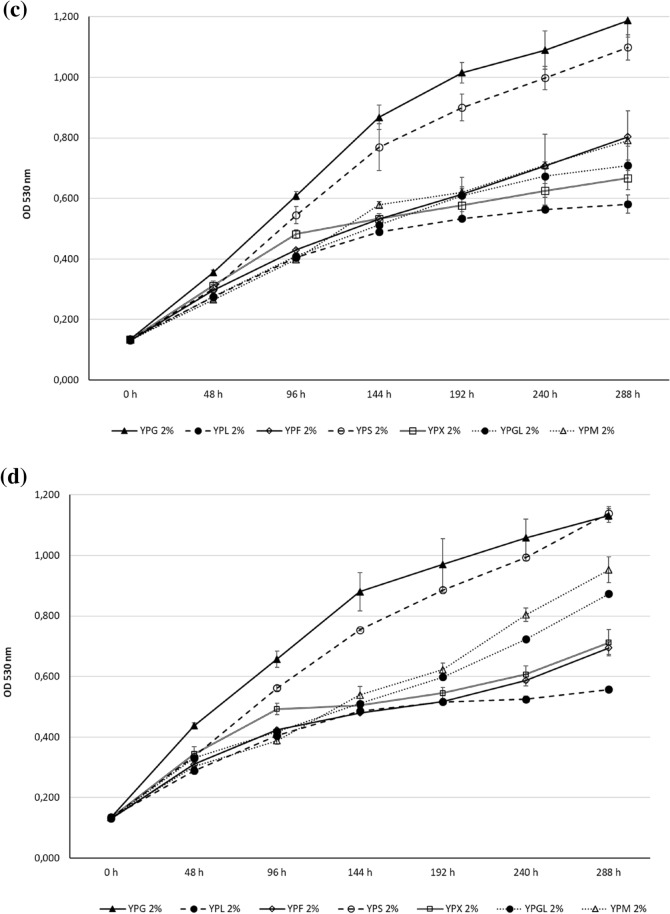

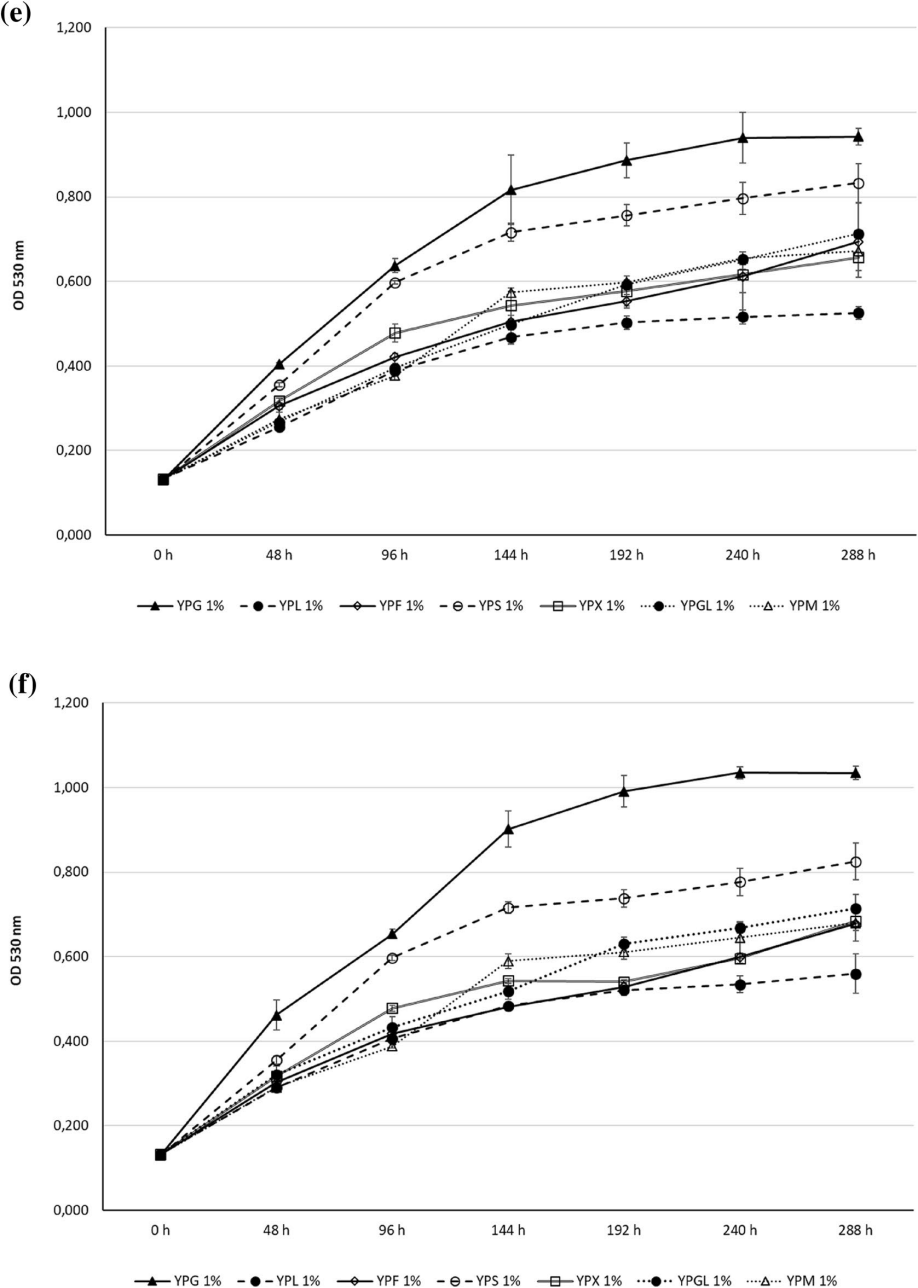
Cell growth ability of the examined black yeasts in liquid media containing different carbon sources (Glucose, Fructose, Lactose, Sucrose, Xylose, Glycerol and Mannitol): **a** *Z. obscura* LS31012019 and **b** *A. pullulans* ATCC 15233 kinetics in 5% YPG, YPF, YPL, YPS, YPX, YPGL, YPM; **c** *Z. obscura* LS31012019 and **d** *A. pullulans* ATCC 15233 kinetics in 2% YPG, YPF, YPL, YPS, YPX, YPGL, YPM; **e** *Z. obscura* LS31012019 and **f** *A. pullulans* ATCC 15233 kinetics in 1% YPG, YPF, YPL, YPS, YPX, YPGL, YPM. Values are expressed as the means ± standard deviations

The growth of *Z. obscura* LS31012019, in comparison to *A. pullulans* ATCC 15233, in presence of different carbon sources was presented in Fig. 4. In general, glucose represents the most suitable carbon source for both the examined black yeasts but the observed growths resulted to be not strictly dose-dependent.

In detail, considering the highest concentration (5%) of each carbon source, the growth peak for *Z. obscura* LS31012019 was observed in YPG after 240 h (OD 1.083 ± 0.043), followed by YPS (OD 1.022 ± 0.083), YPF (OD 0.772 ± 0.046) and YPX (OD 0.766 ± 0.079), then by the others remaining sugars. In the following time point (288 h), a general decrease was evidenced in most of the culture media, with the only exception of YPF (OD 0.801 ± 0.091) (Fig. 4a). In the case of *A. pullulans* ATCC 15233, the growth peak was reached after 192 h in YPG (OD 1.1049 ± 0.053), followed by YPX (OD 0.915 ± 0.031) and YPGL (OD 0.724 ± 0.085); the growth still increased in presence of fructose and sucrose up to 240 h with OD values of 0.953 ± 0.028 and 0.942 ± 0.059 respectively. After 240 h of incubation, a remarkable decrease was observed in YPG, YPX, YPL, YPGL and YPM whilst the OD values continued to increase in YPF and YPS (0.953 ± 0.029 and 0.942 ± 0.06 respectively). In the prolonged time (288 h), the viability of *A. pullulans* ATCC 15233 was subjected to a more evident decrease (Fig. 4b). For both the examined strains, a limited growth was observed with 5% lactose (YPL) and mannitol (YPM).

The growth in presence of 2% carbon sources, revealed that *Z. obscura* LS31012019 as well as *A. pullulans* ATCC 15233 continued to constantly grow in all the examined culture media up to 288 h, without the decline observed in the same media containing a more concentration of carbon source (5%). Indeed, the growth peak for *Z. obscura* LS31012019 was evidenced in YPG (OD 1.188 ± 0.055), followed by YPS (OD 1.099 ± 0.042), YPF (OD 0.803 ± 0.086), YPGL and YPM (OD 0.709 ± 0.017 and 0.791 ± 0.019 respectively), YPX (OD 0.667 ± 0.038). Lower growth was observed in YPL (0.581 ± 0.030) (Fig. 4c). For *A. pullulans* ATCC 15233, similar growth was noticeable in YPG and YPS (OD 1.132 ± 0.022 and 1.139 ± 0.021 respectively); a remarkable growth was detected in YPM with OD 0.953 ± 0.043 and YPGL (OD 0.830 ± 0.012), followed by YPX (OD 0.712 ± 0.043) and YPF (OD 0.695 ± 0.021). Also in this case, the minimum growth was observed in YPL (0.557 ± 0.030) (Fig. 4d).

When *Z. obscura* LS31012019 and *A. pullulans* ATCC 15233 were cultivated in media containing 1% of each carbon source, the growth continued up to 288 h without any evident decrease, as observed with 2%. In the case of *Z. obscura* LS31012019, the growth peak was reached in YPG (OD 0.924 ± 0.019), followed by YPS (OD 0.833 ± 0.046), YPGL (OD 0.712 ± 0.026), YPF (OD 0.694 ± 0.091), YPM and YPX (OD 0.672 ± 0.046 and 0.657 ± 0.047) (Fig. 4e). As regards *A. pullulans* ATCC 15233, a similar trend was observed with the highest growth in YPG (OD 1.034 ± 0.016) followed by YPS (OD 0.825 ± 0.043); noteworthy, the growth in YPGL and YPM reached OD of 0.714 ± 0.032 and 0.680 ± 0.043 respectively, followed by YPX and YPF (OD 6.844 ± 0.022 and 0.680 ± 0.047 respectively) (Fig. 4f). For both the black yeasts, the lowest growth was observed in presence of lactose.

### GSH concentration in of *Z. obscura* LS31012019

The Ellman’s reagent can measure both low-molecular-mass thiols, such as glutathione, and thiol groups on proteins. The glutathione values could be affected by the presence of proteins, but the GSH is the most abundant intracellular thiol, present at millimolar concentrations. In this investigation, the concentration of GSH in *Z. obscura* LS31012019 was 0.95 ± 0.10 mM and in *A. pullulans* ATTC 15233 was 1.53 ± 0.15 mM. Comparing the Glutathione (GSH) levels as µmol/mg, *Z. obscura* LS31012019 and *A. pullulans* showed comparable values of 0.321 ± 0.035 and 0.233 ± 0.021 µmol/mg respectively (Table 3).

**Table 3:** GSH content in *Z. obscura* LS31012019 and *A. pullulans* ATCC 15233 cell lysates

Yeast strains	GSH content (mM)	GSH content (µmol/mg)
*Z. obscura* LS31012019	0.95 ± 0.10	0.321 ± 0.035
*A. pullulans* ATCC 15233	1.53 ± 0.15	0.233 ± 0.021

### Comparative evaluation of pigments from *Z. obscura* LS31012019 and *A. pullulans* ATCC 15233

**Fig. 5:**
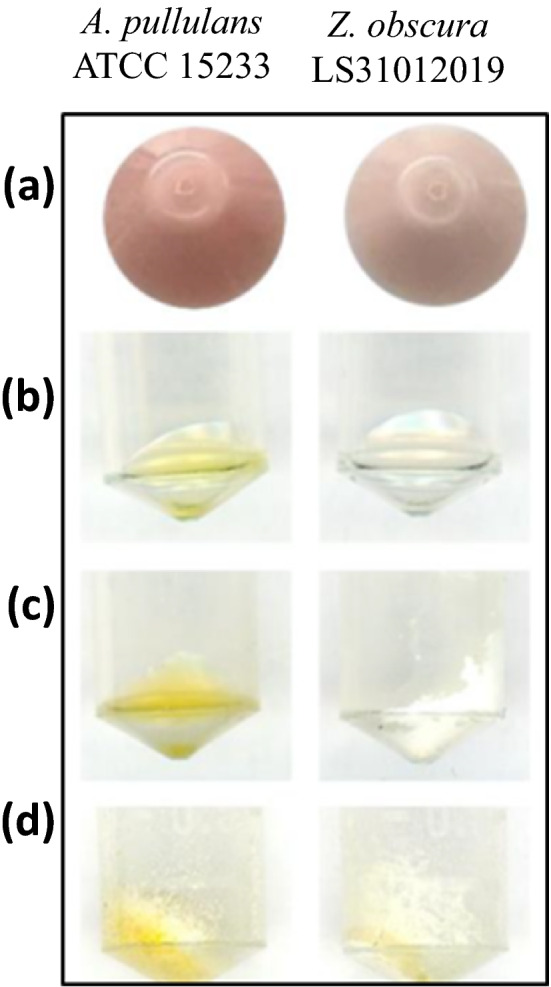
Extraction of pigments with different solvents from lyophilzed cells of *Z. obscura* LS31012019 in comparison to *A. pullulans* ATCC 15233: **a** pellets before chemical extraction; **b** pellets after hexane extraction; **c** pellets after ethanol extraction; **d** pellets after acid ethanol extraction

Even if both *Z. obscura* LS31012019 and *A. pullulans* ATTC 15233 are black yeasts, they do not produce melanin in the applied grow condition (YPM medium) taking on a pinkish colour, as shown in the pellets obtained after centrifugation (Fig. 5a). The extraction of the dried-cell crushed powder with hexane generates a yellow oleaginous pellet in both the examined strains (Fig. 5b), while the following solvent extractions of the powder yielded a yellow solid matrix with a more intense colouration in *A. pullulans* ATTC 15233 powder compared to that of *Z. obscura* LS31012019 (Fig. 5c, d).

## Discussion

To date, very poor information is available in the literature regarding the peculiar biochemical or coltural features of the new specie *Zalaria obscura*. Considering that this black yeast is phylogenetically related to *Aureobasidium pullulans*, a microorganism with a well-defined profile of biotechnological and biocontrol activities (Prasongsuk et al. 2018), our research was focused on better understanding the similarities or the differences between these species to define the possible application of *Z. obscura* for human activity, such as the industrial or the environmental one. Indeed, regarding the new specie *Zalaria*, only four researches are available in the literature and no information on its specific biochemical characteristics are still reported. Our group is focused to improve the knowledge of *Zalaria obscura* and, in this direction, for the first time we present the SDS-PAGE of the isolate *Z. obscura* LS31012019 in comparison to that of *A. pullulans* ATCC 15233. The observed similarities support the data concerning the phylogenetic relationships with *Aureobasiudium* spp., already evaluated by ITS analysis (Humphries et al. 2017), adding novel supporting information on this topic.

The growth ability of *Z. obscura* LS31012019, in comparison to *A. pullulans* ATCC 15233, in liquid media at different temperatures was determined. The best performance was obtained in YPD which contains the same amount of dextrose (20 g/L) as SDB, but also yeast extract (10 g/L) as an available carbon source. In the literature is well reported that YP contains the components available for yeast propagation as well as the biosynthetic building blocks; interestingly, this undefined medium can support the growth of different yeast strains even in absence of other carbon sources, accelerating it with the addition of 20 g/L glucose (Hahn-Hagerdal et al. 2005) as observed in our experiments with YPD. Chemicals and physical parameters affect microbial growth and, in the case of yeasts, the availability of nutrients, as well as temperature and pH, can affect the development of cells. In the presented investigation, the temperature played a key role in the growth ability of *Z. obscura* LS31012019 which reached its optimum at 25 °C, thus showing the typical feature of mesophilic fungi, which have T_opt_ of 25 − 30 °C and generally tolerate a wider range of temperatures and related stress (Abu Bakar et al. 2020). Fungal adaptation to variable temperatures implicates alterations in the utilization of many metabolic pathways to compensate for the amount of energy needed in delivering the stress response. As temperature increases, the metabolism increases as a simply physical consequence, and then rapidly declines at higher temperatures when the metabolic systems start to fail (leading to the eventual cell death). It can be stated that fungi respond to temperature stress through the regulation of various proteins, and many studies have evidenced as important cellular functions are affected, such as energy production and metabolism, oxidative phosphorylation, cell cycle and division, biosynthetic pathways, defence against oxidative and other stresses, and cellular signalling mechanisms (Abu Bakar et al. 2020; Bai et al. 2015; Kostadinova et al. 2011; Yan et al. 2020).

Another important factor affecting yeast growth is represented by carbon sources, elements fundamental for a lot of vital biochemical processes. To better understand the role of carbon sources in *Z. obscura* LS31012019, six sugars, apart from glucose, were added to YP broth at three different concentrations (5%, 2% and 1%). As expected, at all the tested concentrations, glucose was the most suitable carbon source as evident in the highest growth reached in YPG during the experiments. In any case, the behaviour of *Z. obscura* LS31012019 was different from that of *A. pullulans* ATCC 15233; from the data, it can be observed that *Z. obscura* LS31012019 continued to grow at 5% YPG for a longer period (peak at 240 h) compared to *A. pullulans* ATCC 15233 (peak at 192 h), and similarly, the OD value of *Z. obscura* LS31012019 was higher in 2% YPG at 288 h in comparison to that of *A. pullulans* ATCC 15233. One explanation of this behaviour could be that the excess carbon would exhibit an inhibitory effect on cell growth, as reported for *A. pullulans* (Kim et al. 2000; Sheng et al. 2016). On the contrary, at low glucose concentration (1%), the ability to grow was lower in *Z. obscura* LS31012019 compared to *A. pullulans* ATCC 15233, underlying the importance of glucose in the basic metabolic process. Indeed, glucose is the favoured carbon and energy source in yeasts and glucose repression and derepression essentially involved genes for oxidative metabolism and tricarboxylic acid (TCA) cycle, metabolism of alternative carbon sources, or genes for gluconeogenesis. In presence of glucose, it was reported a decrease in transcription or translation at the gene level or an increase in protein degradation at the protein level (Weinhandl et al. 2014). Generally, monosaccharide carbon sources are preferred to disaccharide and polysaccharide carbon sources, because directly available in the biochemical process. The current findings evidenced that sucrose would be degraded to glucose and fructose, and then fructose would be isomerized to glucose (Sheng et al. 2016). Our data support this thesis, and effectively among the examined sugars, sucrose resulted in efficacy support the growth of *Z. obscura* LS31012019 and *A. pullulans* ATCC 15233. For the latter, is well known that it possesses the enzymatic apparatus able to use sucrose for pullulan production (Prasongsuk et al. 2007), but also *Z. obscura* LS31012019 seems to have the ability to degrade sucrose to glucose and fructose, as demonstrated by the high levels of growth in YPS at all the tested concentrations. We can state that, concerning the carbon source concentration, at the highest tested level (5%) the growth of *Z. obscura* LS31012019 (in terms of OD maximum value) was supported by Glucose > Sucrose > Fructose > Xylose > Lactose > Glycerol > Mannose while that of *A. pullulans* ATCC 15233 by Glucose > Sucrose > Fructose > Xylose > Glycerol > Lactose > Mannose. At 2% concentration, some differences arose and it can be observed that the growth of *Z. obscura* LS31012019 was supported by Glucose > Sucrose > Fructose > Glycerol > Mannose > Xylose > Lactose and *A. pullulans* ATCC 15233 by Glucose > Sucrose > Mannose > Glycerol > Xylose > Fructose > Lactose. The lowest tested concentration (1%), supported the growth similarly to 2% concentration in the case of *Z. obscura* LS31012019, following the scheme Glucose > Sucrose > Glycerol > Fructose > Mannose > Xylose > Lactose, but not for *A. pullulans* ATCC 15233 with the sequence Glucose > Sucrose > Glycerol > Mannose > Xylose and Fructose > Lactose. From the data, we can sustain that under similar growth conditions, *Z. obscura* LS31012019 and *A. pullulans* ATCC 15233 utilize similarly the available carbon sources. Indeed, *Z. obscura*, as well as *A. pullulans,* delayed growth in presence of sugar alcohols at high concentrations such as Mannose and Glycerol, but at lower concentrations (2% and 1%), these substrates were effectively utilized to support the growth of both the strains. It’s conceivable that *Z. obscura* LS31012019, as well as *A. pullulans* ATCC 15233, faced off a shift from a fermentative to a nonfermentative mode of growth, with a reprogramming in gene expression pathways (Turcotte et al. 2009).

As mentioned above, glucose and sucrose can directly enter the glycolytic pathway in biochemical processes, but in the absence of these sugars, the examined black yeasts can utilize indifferently the available carbon source to survive, stressing their adaptive ability to the nutritional conditions. Also in this case, the literature reported that *Aureobasidium* spp. produce extracellular enzymes for utilizing different substrates (amylase, proteinase, lipase and estaerase) as well as a series of enzymes specific for carbon sources such as cellulose, lytic polysaccharides monooxygenases, xylose transporter and xylose isomerase, β-fructofuranosidases and so on (Chi et al. 2022). Noteworthy, some of these enzymes can be probably responsible of the observed growth of *Z. obscura* LS31012019 in media containing different available carbon sources, such as fructose, glycerol and xylose. The ability to convert sugars into ethanol is specific to Ascomycetous yeasts (in particular Saccaromycotina), a trait of biotechnological relevance for human activity (Gonçalves et al. 2019), but in the literature, most of the reported studies were focused on pullulan production processes for *Aureobasidium* spp. (An et al. 2017; Cheng et al. 2011; Sheng et al. 2016) and no data are still available on the new specie *Zalaria obscura*. Among the tested carbon sources, only lactose appears to be the least suitable for supporting the growth of both the examined strains, which have probably used the yeast extract present in the medium (YP) to maintain their viability. This possibility was also described by Burgstaller et al. (2022) that reported as the growth of some oleaginous yeast was observed in the respective lactose-containing medium without any decrease in lactose levels as determined by the analytic method. From these observations, we can hypothesize that *Z. obscura* LS31012019, as well as *A. pullulans* ATCC 15233, did not possess the enzymatic apparatus able to biochemically elaborate lactose for further processing pathways. Human activities contribute to the rapid generation of waste products that should be correctly recycled to avoid accumulation and dispersion in the environment. In this regard, ascomycetes can potentially play a crucial role as biocatalysts given their ability to produce enzymes that can break down recalcitrant structures such as industrial and food waste materials (Pandey et al. 2015). Nowadays, mostly filamentous fungi were considered for this application (Ferreira et al. 2016), but the observed ability of *Z. obscura* LS31012019 and *A. pullulans* ATCC 15233 to utilize not only glucose or sucrose but also indifferently the available carbon sources, could be useful to expand the type of species for the treatment of food industry waste, a field where the biological treatment occupies a relevant position.

Glutathione (GSH) is an important antioxidant in prokaryotes and eukaryotes that detoxifies reactive oxygen species (ROS) and is involved in gene expression modulation, redox signalling, and enzymatic activities regulation. It was observed that yeast strains lacking GSH or altered in their GSH redox state are more sensitive to oxidative and environmental stress (Grant 2001; Zechmann et al. 2011). In the most studied yeasts, *Saccharomyces cerevisiae* as well as in non-conventional yeasts, GSH may be involved in basilar cellular functions, such as maintaining mitochondrial and membrane integrity (Pócsi et al. 2004), and also assumes pivotal roles in response to sulfur and nitrogen starvation, detoxifying toxic metabolites, heavy metals and xenobiotics, protecting against oxidative stress, and transitioning into mycelium in *Candida* and *Aureobasidium* spp. (Penninckx 2002). From our data, the recovered GSH content in *Z. obscura* LS31012019, as well as *A. pullulans* ATCC 15233 was lower than that reported for *S. cerevisiae* (up to 10 mM) (Penninckx 2002**)**. This could be probably explained considering that under normal aerobic conditions (as those used in our experiements), glutathione is matnained in the reduced form (GSH), while the exposure to oxidative stress induces a reduction in GSH levels, shifting the redox balance towards the oxidized form (Grant 2001).

Pigmentation is the peculiar characteristic of black yeasts, in particular the one related to melanin. In addition, another type of pigment is present in these microorganisms, known as the carotenoids. They have several functions, such as supporting light-harvesting during photosynthesis, protecting the cell from high light exposure, survive to UV radiation, oxidative stress, and water or salt stress. Thus, carotenoids became interesting for industrial applications, especially for the increasing consumer demand for high-quality and “natural” food nutritional supplements, cosmetics, or other health purposes (Flieger et al. 2018). The biosynthesis of carotenoids begins with the conversion of acetyl-CoA catalyzed by specific enzymes (reductases, kinases, and decarboxylases) to a five-carbon carotenoid precursor, isopentenyl pyrophosphate (IPP). The addition reactions of three IPPs lead to the formation of geranyl–geranyl pyrophosphate (GGPP) and the condensation of two GGPP particles, catalyzed by phytoene synthase, produces phytoene (C40), a precursor that, depending on the type of microorganisms, can be next transformed into carotene, arotene, torulene, lutein, torularhodin, zeaxanthin, and astaxanthin (Mussagy et al. 2019). Indeed, the extraction of pigments, in particular the carotenoids, from these microorganisms can be difficult due to their thick and rigid cell walls. Moreover, chemical and physical parameters affecting the stability of the pigment during extraction such as pH values, oxygen concentration, light and temperature should be considered. Mechanical treatment for cell lysis appears to be the preferred method when handling black yeasts because undamaged cells are necessary for successful pigment extraction since they are susceptible to oxidative stress with the consequent production of ROS. In addition, is advisable to keep the samples at − 4 °C and protect them from light, as far as possible (Craft and Soares 2002). In this direction, in the presented investigation different organic solvents (ethanol and hexane, polar and apolar respectively) coupled with a mechanical method for the pigments extraction from lyophilized cells of *Z. obscura* LS31012019 and *A. pullulans* ATCC 15233 were applied. The preliminary results allowed to obtain pellets with different features and colours between the examined black yeasts. In a particular way, the observed yellowish oleaginous aspect of the pellets after hexane extraction may be due to the presence of carotenoids, a large family of fat-soluble pigments usually composed of eight isoprene units with conjugated double bonds. These molecules can take on yellow, orange, or reddish colours and are ubiquitous in photosynthetic species but are frequently found also in fungi. In some fungal species, the biochemistry and genetics of biosynthesis of carotenoids are well-studied (Avalos and Limón 2015), especially the production of β-carotene, neurosporaxanthin and astaxanthin. This attention may be related to the public demand for natural, eco-friendly and safe pigments for human application worldwide (Lagashetti et al. 2019), for which fungi represent a very interesting source for their fast growth in cheap media, water-independent growth rate, adaptive abilities to many different environmental conditions. Besides the fungal species already used for pigments production (Rapoport et al. 2021), the research is focused to seek new fungal species with specific abilities in pigment production. For *Z. obscura* LS31012019 will be necessary to complete the pigments characterization using different growth conditions and analytic methods, such as HPLC, for a better understanding of pigments type and possible industrial application.

Overall, all the obtained results support the concept that yeasts isolated from natural environments and hostile habitats have an adaptable growth and metabolism in relation to the encountered conditions. In this direction, *Z. obscura* LS31012019, isolated from a harsh environment, owns particular attributes, such as the ability to grow under different culture media and temperatures and utilize carbon sources apart from glucose and sucrose, shifting to a non-fermentative metabolism. Starting from our preliminary but encouraging results, deeper studies are ongoing to verify the applicability of new species of black yeasts in several biotechnological processes, such as the ones for food and industrial waste treatments.

## Data Availability

The datasets generated and/or analyzed during the current study are available from the corresponding author on reasonable request.
